# Simultaneous but independent spatial associations for pitch and loudness

**DOI:** 10.1007/s00426-024-01970-9

**Published:** 2024-05-09

**Authors:** Sarah Koch, Torsten Schubert, Sven Blankenberger

**Affiliations:** Department of Psychology, Martin Luther University Halle-Wittenberg, Halle (Saale), Germany

## Abstract

For the auditory dimensions loudness and pitch a vertical SARC effect (Spatial Association of Response Codes) exists: When responding to loud (high) tones, participants are faster with top-sided responses compared to bottom-sided responses and vice versa for soft (low) tones. These effects are typically explained by two different spatial representations for both dimensions with pitch being represented on a helix structure and loudness being represented as spatially associated magnitude. Prior studies show incoherent results with regard to the question whether two SARC effects can occur at the same time as well as whether SARC effects interact with each other. Therefore, this study aimed to investigate the interrelation between the SARC effect for pitch and the SARC effect for loudness in a timbre discrimination task. Participants (*N* = 36) heard one tone per trial and had to decide whether the presented tone was a violin tone or an organ tone by pressing a top-sided or bottom-sided response key. Loudness and pitch were varied orthogonally. We tested the occurrence of SARC effects for pitch and loudness as well as their potential interaction by conducting a multiple linear regression with difference of reaction time (dRT) as dependent variable, and loudness and pitch as predictors. Frequentist and Bayesian analyses revealed that the regression coefficients of pitch and loudness were smaller than zero indicating the simultaneous occurrence of a SARC effects for both dimensions. In contrast, the interaction coefficient was not different from zero indicating an additive effect of both predictors.

## Introduction

Several stimulus dimensions show a horizontal, spatial association (Macnamara et al., 2018). Probably one of the most prominent examples are numbers: Small numbers are assumed to be represented left while large numbers are assumed to be represented right on a spatial mental number line (Dehaene et al., 1993; Feigenson et al., 2004; Restle, 1970). Empirical evidence for this assumption stems from the SNARC effect (Spatial-Numerical Association of Response Codes) first investigated by Dehaene et al. (1993). In that study, participants decided whether a presented number was odd or even by pressing a left-sided or right-sided response key. Participants responded faster to small numbers with the left-sided response key compared to responding with a right-sided response key and vice versa for large numbers. In recent decades, a comparable effect has been found for other stimulus dimensions which lead to the general term SARC effect (Spatial Association of Response Codes, Macnamara et al., 2018).

Another term widely used is the abbreviation SQUARC effect (Spatial-Quantity Association of Response Codes) introduced by Walsh (2003) in the proposal of A Theory of Magnitude (ATOM; Bueti & Walsh, 2009). According to ATOM, the three domains of time, space, and quantity are represented on a common cortical metric, which can also be interpreted as a generalized magnitude representation system (Bonn & Cantlon, 2012). An important prediction of ATOM is the existence of spatial associations for each magnitude dimension. This association is reflected in the SQUARC effect, that is, shorter reaction times to small quantities with a left-sided response than with a right-sided response and vice versa for large quantities (Walsh, 2003, 2015).

In addition, SARC effects have also been shown for the auditory dimensions loudness and pitch. In general, participants respond faster to soft or low tones with a left-sided response key compared to a right-sided response key and vice versa for loud or high tones (Fairhurst & Deroy, 2017; Guilbert, 2020; Hartmann & Mast, 2017; Lega et al., 2020; Lidji et al., 2007; Rusconi et al., 2006). The SARC effects for loudness and pitch also occur in the vertical dimension indicating that soft or low tones are associated with the spatial information ‘bottom’ while loud or high tones are associated with the spatial information ‘top’ (Bruzzi et al., 2017; Fernandez-Prieto et al., 2017; Lega et al., 2020; Lidji et al., 2007; Pitteri et al., 2017; Rusconi et al., 2006). This is in line with the observation, that spatial associations for different stimulus dimensions can occur in several spatial axes such as vertical or radial (i.e. near-far) axes (see Winter et al., 2015 for a review).

SARC effects for pitch and loudness are typically explained by assuming a spatial representation of the corresponding auditory dimension. However, the structure of the assumed spatial representation differs between pitch and loudness. Pitch is assumed to be represented on a two-dimensional spatial helix structure (Shepard, 1982; Ueda & Ohgushi, 1987). Contrary, the SARC effect for loudness is explained by loudness being represented as a magnitude dimension with a linear spatial association (e.g. Bruzzi et al., 2017). This indicates that the effects rely on similar mechanisms but separated representations with different spatial structures. However, little is known how these assumed separated representations relate to each other, that is whether the SARC effects for pitch and loudness can occur simultaneously and independently from each other. Therefore, this study aimed to investigate the interrelation between the SARC effects for pitch and for loudness. For this, we tested whether both SARC effects interact with each other, which would be reflected in a larger SARC effect for pitch for loud tones compared to soft tones and a larger SARC effect for loudness for high tones compared to low tones. For a better distinction between both effects, we will refer to the effects as SPARC effect (Spatial-Pitch Association of Response Codes, Lidji et al., 2007) and as SLARC effect (Spatial-Loudness Association of Response Codes).^1^

Before outlining the assumed interrelation in more detail, we will first describe the main findings for the SPARC and SLARC effects, separately. The SPARC effect depends on the interplay of various factors, namely, musical experience of the participants, the spatial arrangement of response keys, and whether pitch is the task relevant dimension or not. When participants have to classify the pitch of a presented tone relative to a standard pitch, a SPARC effect occurs regardless of the spatial arrangement of the response keys and musical experience (Guilbert, 2020; Lega et al., 2020; Lidji et al., 2007; Rusconi et al., 2006). In contrast, when pitch is not relevant for the task and participants have to respond to another attribute of the tone, for example its timbre, non-musicians show only a SPARC effect with vertically but not with horizontally aligned response keys. Contrary, musicians still show a SPARC effect in a timbre discrimination task in the horizontal dimension as well as in the vertical dimension (Lega et al., 2020; Lidji et al., 2007; Rusconi et al., 2006).

The occurrence of the vertical SPARC effect is typically explained by the assumption of a mental spatial representation of pitch on a bottom-to-top helix structure (Shepard, 1982; Ueda & Ohgushi, 1987). Low pitches are assumed to be represented bottom while high pitches are assumed to be represented top. Additionally, the assumed representation model takes into account that the relationship between the physical dimension frequency and the perception of pitch is non-linear: An increasing frequency does not only lead to the impression of an increasing pitch height but does also change the perceived quality of the tone. This is referred to as pitch chroma and is indicated by the circular organization of tones within a helix plane. The occurrence of the vertical SPARC effect even under conditions in which pitch is not relevant for the task further indicates that the spatial information is automatically co-activated comparable to the automatic activation in case of the SNARC effect in a parity judgment task (Dehaene et al., 1993), which is in line with the assumption of an innate spatial representation of pitch. Note that an alternative explanation could be the semantic overlap between the response codes and the stimulus codes, as pitch is described in spatial terms in some languages. However, the SPARC effect also occurs in participants whose native language does not describe pitch via spatial terms (Fernandez-Prieto et al., 2017), thus invalidating the semantic overlap explanation.

Contrary to the SPARC effect, the SLARC effect does not dependent on the arrangement of response keys or the relevance of loudness for the task. Although earlier studies did not find a SLARC effect in the horizontal dimension (Ren et al., 2011), the effect was later found by other studies (Chang & Cho, 2015; Fairhurst & Deroy, 2017; Hartmann & Mast, 2017). Furthermore, several studies found the SLARC effect in the vertical dimension (Bruzzi et al., 2017; Fernandez-Prieto et al., 2017). The SLARC effect is not only present when participants have to judge the loudness of a tone relative to a standard tone (Bruzzi et al., 2017; Hartmann & Mast, 2017) but does also occur in timbre discrimination tasks with horizontally (Chang & Cho, 2015) and vertically arranged response keys (Koch et al., 2023). This indicates an automatic activation of the spatial information of loudness comparable to the automatic activation of the spatial information in the case of pitch.

Typically, the SLARC effect is explained by assuming that loudness is represented as a magnitude according to ATOM (Bueti & Walsh, 2009; Walsh, 2003), as suggested for instance by Bruzzi et al. (2017). The SLARC effect found in previous studies is in line with this prediction as loud tones, that is tones with high intensity, are associated with right and soft tones associated with left (Hartmann & Mast, 2017). Furthermore, ATOM predicts interactions between magnitude dimensions (Walsh, 2003, 2015) and several studies found that loudness interacts with other magnitude dimensions like numerical magnitude (Alards-Tomalin et al., 2015; Hartmann & Mast, 2017; Heinemann et al., 2013) or physical size (Smith & Sera, 1992; Sutherland et al., 2014; Takeshima & Gyoba, 2013) which also supports the assumption that loudness is represented as a magnitude. Furthermore, loudness is a prothetic or quantitative dimension (Stevens, 1957; Stevens & Galanter, 1957), which is an important theoretical prerequisite for a dimension to be considered part of ATOM (Walsh, 2003, 2015). Taken together, assuming that loudness is represented as a magnitude in the sense of ATOM, the SLARC effect could be considered as an instance of the general SQUARC effect. This assumption is also supported by the notion that the SLARC effect seems to be continuous (Koch et al., 2023) which contradicts the most prominent alternative explanation, namely the polarity correspondence principle (Chang & Cho, 2015; Proctor & Cho, 2006).

So far, empirical evidence suggests that the SPARC and SLARC effects are due to two different spatial representations but a direct empirical test of this assumption is still missing. In addition, previous studies investigating spatial associations for spoken number words have found that the SPARC and SLARC effects interact with the already mentioned SNARC effect in a way that contradicts several theoretical assumptions. Numbers are assumed to be represented as a magnitude in terms of ATOM (Bueti & Walsh, 2009; Walsh, 2003, 2015) and therefore would share a magnitude representation with loudness. Pitch, on the other hand, is explicitly excluded from the conceptualization of ATOM because it is a metathetic or qualitative dimension (Stevens, 1957; Stevens & Galanter, 1957). Based on these assumptions, two interaction patterns between the SLARC, SPARC, and SNARC effects are plausible. First, from the premise that an interaction between spatial associations indicates a common origin, one would expect that the SLARC and SNARC effects should interact. This interaction could be reflected in a larger SLARC effect for large spoken numbers compared to small numbers as well as a larger SNARC effect for loud spoken number words compared to soft spoken number words. The SPARC and SNARC effects should be independent of each other. Alternatively, since a shared representation does not rule out purely additive effects (Sternberg, 1969), a second possible scenario could be that loudness and numbers share a common representation, but that the SLARC and SNARC effect simply do not interact. Importantly, the SPARC and SNARC effects should still not interact. Results from previous studies contradict both scenarios: While the SLARC effect does not interact with the SNARC effect (Hartmann & Mast, 2017), the SPARC effect does interact with the SNARC effect (Fischer et al., 2013; Weis et al., 2015, 2016).

In a study by Hartmann and Mast (2017), participants heard spoken number words and had to classify the numerical value, loudness level, or parity. There was no interaction between the SPARC effect and the SLARC effect. Additionally, both effects were limited to the dimension-related task. The SNARC effect only occurred in the parity and number judgment task while the SLARC effect was limited to the loudness judgment task. However, both effects are known to occur even when number magnitude or loudness are irrelevant (Chang & Cho, 2015; Fias, 2001; Koch et al., 2023; for a review for the SNARC effect see Wood et al., 2008). This raises the question of whether two SARC effects can generally occur simultaneously.

Indeed, studies that found an interaction between the SPARC effect and the SNARC effect also found a simultaneous occurrence of both effects (Weis et al., 2015, 2016). However, the results are not entirely consistent, which might be due to different experimental setups. For example, Fischer et al. (2013) investigated the SPARC effect and the SNARC effect in a pitch discrimination task and a number discrimination task with diagonally arranged response keys. Both effects only occurred when the corresponding dimension was task-relevant. Crucially, there was a reversed SNARC effect for the SPARC incompatible trials but not for the SPARC compatible trials. In the studies conducted by Weis and colleagues (Weis et al., 2015, 2016), participants had to classify either the numerical value, pitch, or parity. Participants responded faster in SNARC compatible and SPARC compatible trials compared to incompatible trials regardless of the task. Furthermore, there was a significant interaction between SPARC compatibility and SNARC compatibility. The authors concluded that both effects share a common automatic decision mechanism and further suggested that this mechanism might be based on a common representation of pitch and numbers in the sense of ATOM (Weis et al., 2016). However, as already mentioned, pitch is explicitly excluded from the conceptualization of ATOM due to its metathetic or qualitative characteristic (Walsh, 2003, 2015). From a theoretical point of view, the interaction between SPARC and SNARC compatibility cannot be explained in terms of a common representation in the sense of ATOM.

Taken together, previous findings contradict predictions regarding potential interactions between SARC effects. In addition, it is unclear under which circumstances and for which dimensions two SARC effects can occur simultaneously. Therefore, the aim of our study was twofold. First, we wanted to investigate whether the SLARC effect occurs simultaneously with another spatial association, namely the SPARC effect. We used a timbre discrimination task in which participants had to decide whether a single tone was a violin tone or an organ tone while pitch and loudness were varied orthogonally. Neither loudness nor pitch were relevant for the task, which allowed us to investigate whether both effects would show an automatic, simultaneous occurrence. Furthermore, timbre is equally strong related to both task-irrelevant dimensions, which is not the case for parity as used in previous studies (Hartmann & Mast, 2017; Weis et al., 2015, 2016), which is stronger related to the numerical value than to pitch or loudness. As a second aim, we wanted to test whether the interaction between the SPARC and SNARC effects (Weis et al., 2015, 2016) generalizes to the interrelation between the SLARC and the SPARC effects. The interaction between the SPARC and SNARC effects is explained by a shared representation according to ATOM (Weis et al., 2016). If this is the case, then the interaction should generalize to other magnitude dimensions as well. The SLARC effect is explained by an assumed magnitude representation of loudness (Bruzzi et al., 2017), and therefore one would also expect an interaction between the SLARC effect and the SPARC effect. This would be reflected in a larger SPARC effect for loud tones compared to soft tones as well as a larger SLARC effect for high tones compared to low tones.

To test the simultaneous occurrence as well as a possible interaction between both effects, we conducted a multiple linear regression, with the difference of reaction time between top-sided and bottom-sided responses (dRT) as dependent variable and loudness and pitch as predictors (Fias et al., 1996). We predicted that the SPARC effect and the SLARC effect would occur simultaneously indicated by a negative regression coefficient for loudness as well as for pitch. Previous studies found that SARC effects for two prothetic dimensions did not occur simultaneously (Hartmann & Mast, 2017; Vellan & Leth-Steensen, 2022; Weis et al., 2018) whereas SARC effects for one prothetic and one metathetic dimension occured simultaneously (Weis et al., 2015, 2016). Because pitch and loudness are regarded as metathetic and prothetic dimensions, respectively (Stevens & Galanter, 1957), SARC effects for both dimensions should occur at the same time. With regard to our second research aim, we predicted that if an interaction between both effects occurred, it should be reflected in larger dRT differences between soft and loud tones which are high in pitch compared to tones which are low in pitch.

## Methods

The study’s design as well as our hypothesis and analysis plan were preregistered on aspredicted.org (https://aspredicted.org/CG8_V9Q).

### Participants

*N* = 38 healthy students from Martin Luther University Halle-Wittenberg participated for course credit. Age was restricted to a range from 18 to 35 years to ensure that age-related hearing impairments were negligible between participants. Professional musicians (i.e. people who study or have studied music or work full-time or part-time as musician) were not allowed to participate in the study. The planned sample size was *N* = 36 based on a power analysis for a one-tailed one-sample *t*-test with a significance level of $\alpha =0.05$, Power $1-\beta =0.9$ and a medium effect size of ${d}_{z}=-0.5$. After we reached our determined sample size, we checked the mean error rate and excluded two participants because their error rate exceeded 15%. In line with our preregistration, we collected data from two additional participants. Therefore, data from *N* = 36 participants (*N* = 31 female) were included in the final data analysis. The mean age was *M* = 21.8 years (*SD* = 3). *N* = 33 participants reported being right-handed. All participants reported having normal or corrected-to-normal vision and no participant reported any hearing impairments. Informed written consent was obtained from all individual participants included in the study.

### Materials

The auditory material consisted of 50 different tones with five pitch levels, five loudness levels, and two timbres. Pitch was operationalized by frequency in Hertz and pitch levels ranged from from 261 Hz (F#3) to 523 Hz (C5) with 3 half-tone steps between each pitch level. Loudness levels ranged from 45 to 65 phon with 5 phon between each level. All pitch-loudness combinations were realized as violin tone and as organ tone. Auditory stimuli were first synthesized with the software LMMS (Version, 1.2.0, Junghans & Giblock, 2019). Pitch levels of the tones were then analyzed via spectral analyses in Audacity (Version 3.2.0, The Audacity Team, 2019) and adjusted to the aimed fundamental frequency if necessary. Because the subjective loudness perception depends on sound pressure level and frequency (Fletcher & Munson, 1933), we controlled the loudness level for each frequency as followed. First, we adjusted the sound pressure level for each pitch level according to the isophone curves from the ISO norm for pure tones (International Organization for Standardization, 2003). Afterwards, tones were presented to *N* = 4 independent participants, who adjusted the amplitude of the tones according to their subjective loudness impression, so that all tones from one loudness level sounded equally loud to them regardless of pitch and timbre. Tone presentation and amplitude adjustment was done directly in Audacity. Participants could repeat the tones and adjust the amplitude of each tone as often as they wished. Each final sound file had a sample rate of 48 kHz and a duration of 800 ms.

In the experiment, tones were presented via headphones (Sennheiser HD471). Participants responded to the tones by pressing a top-sided or bottom-sided response key, which were vertically aligned on a custom-built response box. Keys were 1 cm and 16 cm above the table surface. Keys were vertically aligned because several studies found the SPARC and SLARC effects occur for non-musicians in a timbre discrimination task with vertically aligned response keys (Koch et al., 2023; Lega et al., 2020; Lidji et al., 2007; Rusconi et al., 2006). Stimulus presentation and data recording was realized via the software PsychoPy (Version 2021.2.3, Peirce et al., 2019).

### Procedure

Each trial started with the presentation of a fixation cross in the middle of the screen for 500 ms. After a foreperiod of 500 ms, the tone was presented for a maximum of 800 ms or until participants made a response. Because there was no time window, participants could press the response key after the tone ended. After participants gave their response, an inter-trial interval (ITI) of a uniformly distributed duration between 1 and 2 s was presented before the next trial started. Participants were instructed to classify the presented tone as violin tone or organ tone as fast and accurate as possible by pressing the top-sided or bottom-sided response key. They were not told that the tones had different loudness and pitch levels but to make them familiar with the auditory material, participants heard the combinations of the loudest, softest, highest, and lowest tones in both timbres during the instruction phase.

Response mapping was varied within participants and between four sessions. Order of response mapping was counterbalanced across participants. Half of the participants responded in the first and second session with the top-sided response key to the violin tone and with the bottom-sided response key to the organ tone and vice versa in the third and fourth session. The other half of participants had the opposite order of response mapping. Key-hand mapping was also varied between participants. Half of the participants pressed the top-sided response key with their right thumb and the bottom-sided response key with their left thumb, the other half had the opposite mapping. Participants were randomly assigned to one of the four condition groups with *N* = 9 participants in each group.

In each session, all 50 tones were presented 12 times resulting in 600 experimental trials divided into 12 blocks à 50 experimental trials plus three warm-up trials in the beginning of each block. Participants performed 50 training trials in the beginning of each session in which they received feedback about their reaction time and correctness of the response. Each session took between 45 and 50 min.

At the end of the fourth session, participants completed two subscales from the German version of the Goldsmith’s Musical-Sophistication Index (Gold-MSI, Müllensiefen et al., 2014; Schaal et al., 2014). Although professional musicians were not allowed to participate in the study, we assumed that some participants might be familiar with playing an instrument. Some studies rely on cut-off criteria to define non-musicality, for example not playing an instrument for a certain amount of years (e.g. Lidji et al., 2007; Weis et al., 2016). However, these cut-off criteria are often not theoretical or empirical justified. Therefore, we assessed musical experience with a standardized questionnaire to investigate potential influences on the SPARC effect or SLARC effect. For our study, we used the subscales “perception” (9 items) and “musical training” (7 items) with a Cronbach’s $\alpha$ of $\alpha$ = 0.8 for the perception scale and $\alpha$ = 0.91 for the musical training scale ($\alpha$ = 0.83 and $\alpha$ = 0.88 in the study of Schaal et al. (2014), respectively).

### Data analysis

We used R (Version 4.1.2; R Core Team, 2021) and the R-packages *afex* (Version 1.1.1; Singmann et al., 2022), *BayesFactor* (Version 0.9.12.4.4; Morey & Rouder, 2022), *papaja* (Version 0.1.1; Aust & Barth, 2022), and *tidyverse* (Version 1.3.1; Wickham et al., 2019) for all our analyses. Reaction times from incorrect trials (6.9%) were discarded from further analyses. We calculated the trimmed mean reaction times with a trimming amount of 20% (Rosenberger & Gasko, 1983) and the mean error rate for each participant and within-subject condition. In addition, we calculated the mean difference of reaction times ($dRT={RT}_{top}-{RT}_{bottom}$) for each participant, loudness, frequency, and timbre (Fias et al., 1996). For the ANOVA results, *p*-values corrected according to Geisser-Greenhouse (GG) will be reported in case of violations of the sphericity assumption. For the dRT analyses, we will report results from frequentist and Bayesian analyses.

## Results

### ANOVA results

**Fig. 1:**
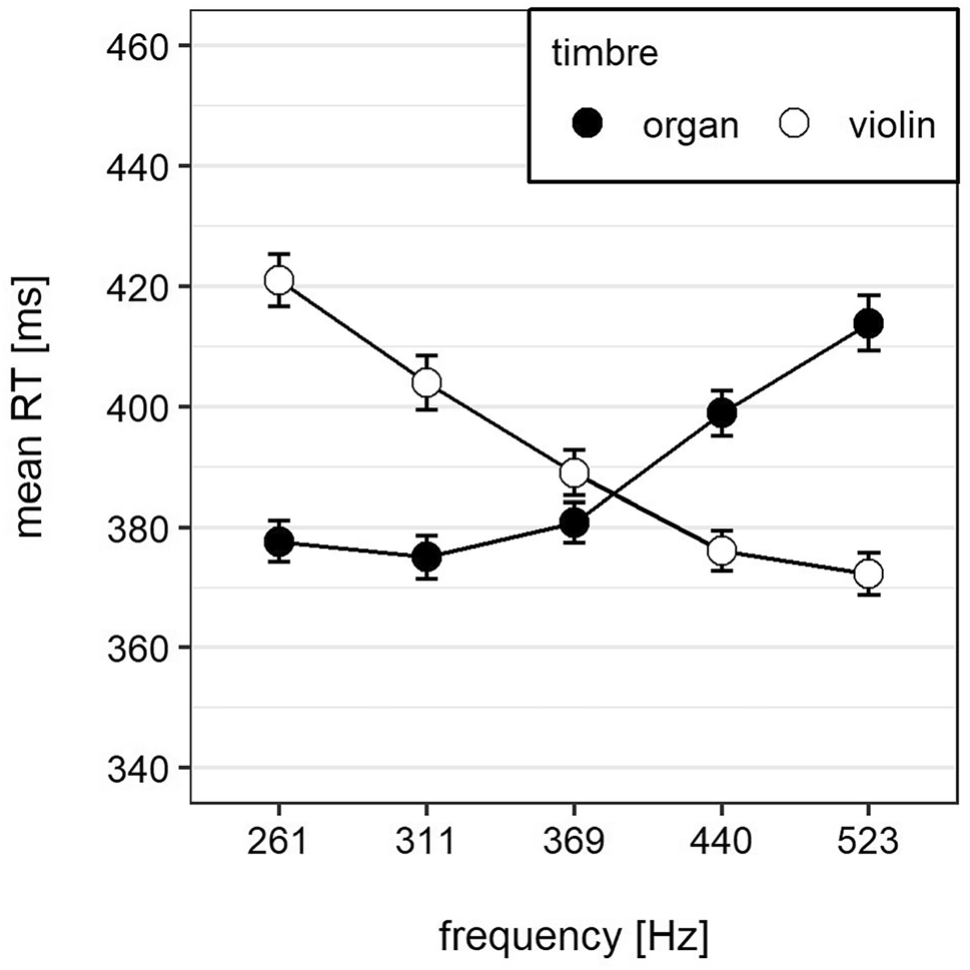
Mean reaction time as a function of frequency and timbre. Error bars represent 95% within-subjects CI (Morey, 2008)

In a first step, we conducted a mixed 5 (loudness) $\times$ 5 (frequency) $\times$ 2 (timbre) $\times$ 2 (response side) $\times$ 2 (order of response side) $\times$ 2 (key-hand mapping) ANOVA with order of response mapping and key-hand mapping as between-subjects factors and the remaining factors as within-subjects factors. Overall, mean reaction time decreased with increasing loudness with 402 ms, 393 ms, 389 ms, 385 ms, and 384 ms from softest to loudest loudness level. This main effect of loudness was significant, $F\left(\mathrm{4,128}\right)=44.14$, $MSE=1211.94$, $p<0.001$ (GG), $\hat{\eta }_G^2 = 0.007$. There was also a significant main effect of frequency, $F\left(\mathrm{4,128}\right)=22.16$, $MSE=1807.04$, $p<0.001$ (GG), ${\widehat{\eta }}_{G}^{2}=0.004$: Participants responded fastest to tones with a frequency of of 369 Hz (385 ms) and their reaction time increased with decreasing (389–399 ms) and increasing frequency (387–393 ms). Additionally, there was also a significant interaction between frequency and timbre, $F\left(\mathrm{4,128}\right)=81.16$, $MSE=7074.73$, $p<0.001$ (GG), ${\widehat{\eta }}_{G}^{2}=0.040$. Participants responded faster to high violin and low organ tones compared to low violin tones and high organ tones (see Fig. 1).

**Fig. 2:**
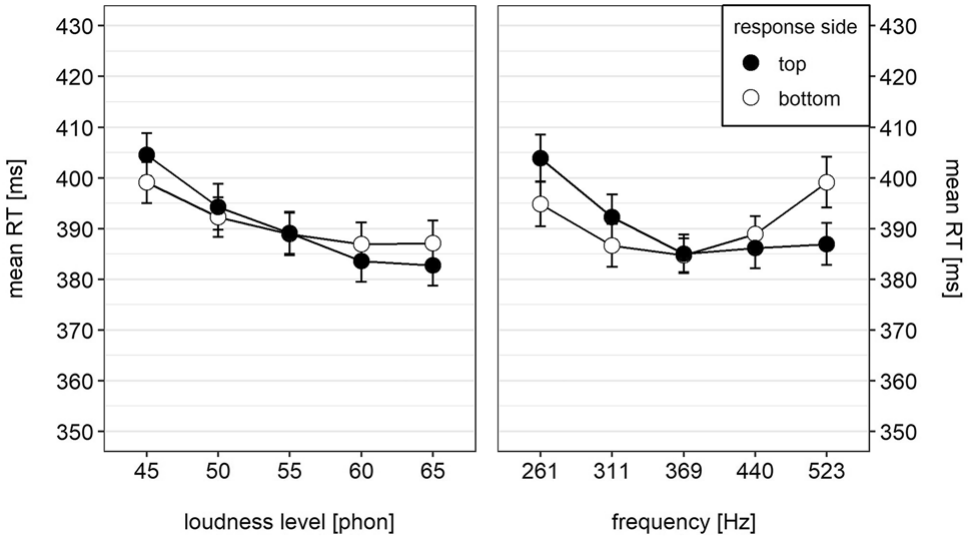
Mean reaction time as a function of response side and loudness (left panel) and response side and pitch (right panel). Error bars represent 95% within-subjects CI (Morey, 2008)

With regard to our hypothesis, there was a significant interaction between loudness and response side, $F\left(\mathrm{4,128}\right)=5.07$, $MSE=697.48$, $p=0.002$ (GG), ${\widehat{\eta }}_{G}^{2}=0.001$ as well as a significant interaction between frequency and response side, $F\left(\mathrm{4,128}\right)=12.07$, $MSE=1988.93$, $p<0.001$ (GG), ${\widehat{\eta }}_{G}^{2}=0.002$. Both interactions are depicted in Fig. 2. Participants responded faster to soft or low tones with the bottom-sided response key compared to the top-sided response key. The opposite holds true for loud and high tones, respectively. For both dimensions, there was also a significant three-way interaction with timbre, with $F\left(\mathrm{4,128}\right)=3.04$, $MSE=848.39$, $p=0.037$ (GG), ${\widehat{\eta }}_{G}^{2}=0.000$ for the loudness $\times$ response side $\times$ timbre interaction and $F\left(\mathrm{4,128}\right)=3.49$, $MSE=670.29$, $p=0.017$ (GG), ${\widehat{\eta }}_{G}^{2}=0.000$ for the frequency $\times$ response side $\times$ timbre interaction. A more detailed analysis of both effects is carried out in the dRT analyses in the next section.

Response side interacted with both between-subject factors, order of response mapping, $F\left(\mathrm{1,32}\right)=5.28$, $MSE=\mathrm{3,309.02}$, $p=0.028$, ${\widehat{\eta }}_{G}^{2}=0.001$, and key-hand-assignment, $F\left(\mathrm{1,32}\right)=6.14$, $MSE=3309.02$, $p=0.019$, ${\widehat{\eta }}_{G}^{2}=0.001$. Participants who responded in the first two sessions to the violin tone with the top-sided response key were in general faster when responding with the top-sided response key (393 ms vs. 396 ms). Contrary, participants who started with the opposite mapping were in general faster when they pressed the bottom-sided response key compared to a top-sided response key (385 ms vs. 389 ms). Additionally, participants who pressed the bottom-sided response key with their right thumb were faster when pressing the bottom-sided response key compared to the top-sided response key (388 ms vs. 393 ms) and vice versa for participants who had the opposite key-hand mapping (387 ms vs. 392 ms). Because 33 out of 36 participants reported being right-handed, this interaction probably reflects the effect of the dominant hand on reaction times. There was also a significant interaction between timbre, response side, and order of response mapping, $F\left(\mathrm{1,32}\right)=31.22$, $MSE=\mathrm{24,385.61}$, $p<0.001$, ${\widehat{\eta }}_{G}^{2}=0.034$. Participants were in general faster in the response mapping of the third and fourth sessions compared to the response mapping of the first and second session, which lead to the significant three-way interaction. Lastly, the three-way interaction between response side, frequency, and order of response mapping was significant, $F\left(\mathrm{4,128}\right)=4.21$, $MSE=1988.93$, $p=0.019$ (GG), ${\widehat{\eta }}_{G}^{2}=0.001$. All other main effects and interactions were non-significant, *p* > 0.05.

### Results from dRT analyses

**Fig. 3:**
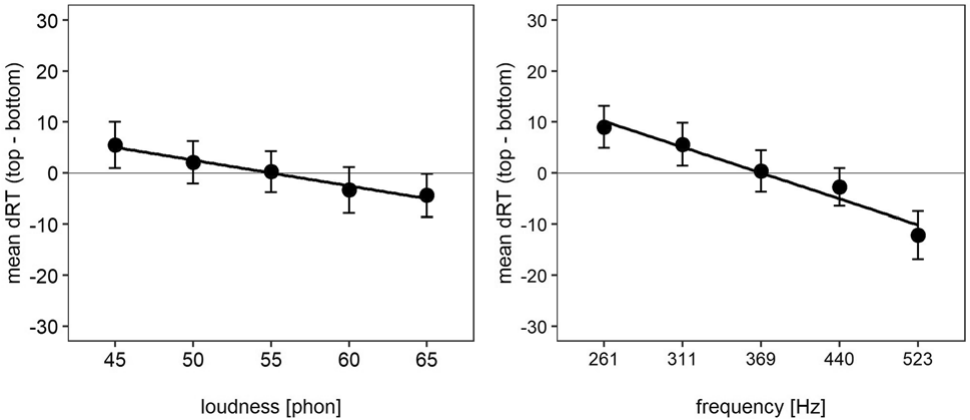
Mean dRT and regression line as a function of loudness (left panel) and frequency (right panel) averaged over the other dimension. Error bars represent 95% within-subjects CI (Morey, 2008)

**Fig. 4:**
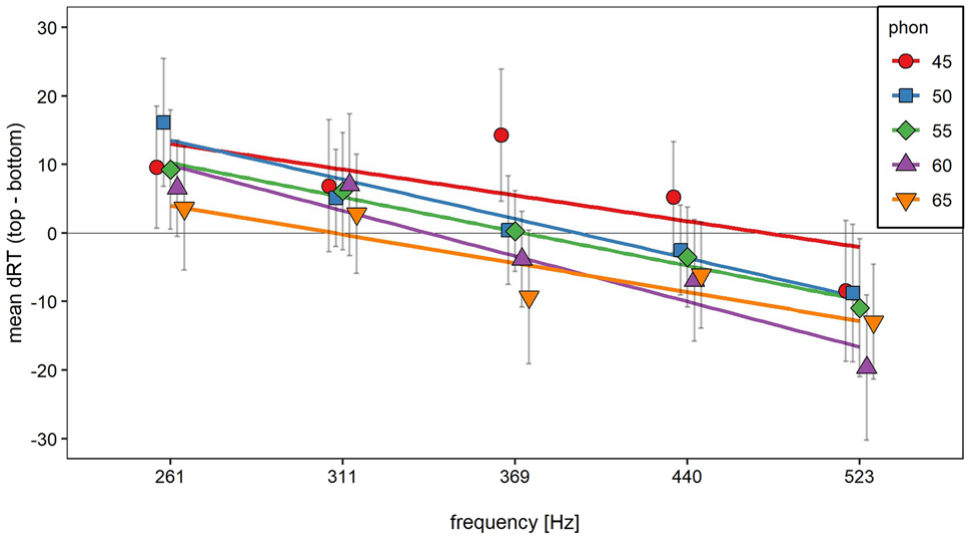
Mean dRT and regression lines as a function of loudness and frequency. Error bars represent 95% within-subjects CI (Morey, 2008)

Mean dRT as a function of loudness and frequency averaged across the other predictor is illustrated in Fig. 3. For both dimensions, mean dRT decreased with an increasing value of the predictor. This holds true when visualizing the relationship between dRT and the loudness $\times$ frequency interaction (see Fig. 4). Mean dRT decreased with increasing loudness level for all frequencies and mean dRT decreased with increasing frequency for all loudness levels. From a visual inspection, there was no clear interaction trend visible.

To test the main and interaction effects, we conducted a multiple linear regression with loudness and frequency as predictors and dRT as dependent variable. We allowed the predictors to interact, although the non-significant interaction between response side, loudness, and frequency already indicated an additive effect. Because of the different scales for loudness and frequency, we first standardized the predictors and the dependent variable to facilitate comparisons between the regression coefficients. Regression coefficients were estimated separately for each participant and timbre and then tested against zero via one-sample *t*-tests (Lorch & Myers, 1990). We used one-tailed (preregistered), one-sample *t*-tests for the main effects and two-tailed (preregistered), one-sample *t*-test for the interaction coefficients. Because timbre significantly interacted with several factors in the previously conducted ANOVA, we followed our preregistration protocol and tested the overall regression coefficients as well as regression coefficients separated for both timbres. This procedure lead to nine one-sample *t*-tests in total. To avoid multiple comparison problems, we conducted a sequential Bonferroni correction, namely Rom’s procedure to adjust significance levels (Olejnik et al., 1997). Results of the *t*-tests as well as the adjusted significance levels are depicted in Table 1. Regression coefficients of the interaction term were not significantly different from zero in all three conditions. The regression coefficients for frequency were significantly smaller than zero in all three conditions. Results for the regression coefficients for loudness were mixed. While in the overall and organ condition, regression coefficients were significantly smaller than zero, this was not the case in the violin condition.

**Table 1:** Results from nine one-sample *t*-tests and corrected significance levels for the overall condition as well as separated for each timbre

Condition	Predictor	*M*	95% CI	t (35)	*p*	$\alpha_{corrected}$
Violin	Interaction	0.01	[− 0.04, 0.05]	0.30	0.768	0.05
Overall	Interaction	− 0.01	[− 0.06, 0.04]	− 0.32	0.749	0.02
Organ	Interaction	− 0.02	[− 0.05, 0.02]	− 0.99	0.330	0.02
Violin	Loudness	− 0.06	[− ∞, − 0.01]	− 2.10	0.021	0.01
Organ	Loudness	− 0.07	[− ∞, − 0.03]	− 3.03	0.002	0.01
Overall	Loudness	− 0.12	[− ∞, − 0.06]	− 3.19	0.001	0.01
Organ	Frequency	− 0.12	[− ∞, − 0.06]	− 3.46	0.001	0.01
Overall	Frequency	− 0.25	[− ∞, − 0.14]	− 3.90	< 0.001	0.01
Violin	Frequency	− 0.14	[− ∞, − 0.08]	− 3.92	< 0.001	0.00

Results from the frequentist analysis indicate that there was no significant interaction between loudness and frequency. To further support the interpretation of an absent effect, we also conducted Bayesian one-sample *t*-tests with a Cauchy prior distribution with a prior width of $r=1/\sqrt{2}$ (Rouder et al., 2009). To resemble the preregistered one-tailed and two-tailed testing, we used a truncated distribution for testing the regression coefficients of loudness and frequency, and the full distribution for testing the interaction coefficients (for a detailed description of this approach, see Morey & Rouder, 2011). Due to multiple testing, we corrected the prior probability of the ${H}_{0}$ according to Westfall et al. (1997) leading to $p\left({H}_{0i}\right)={0.5}^{1/3}=0.79$ for two-sided tests and $p\left({H}_{0i}\right)=1/{3}^{1/6}=0.83$ for one-sided tests.^2^

Mean regression coefficients as well as the corresponding Bayes factors ($B{F}_{10}$ for two-sided, $B{F}_{-0}$ for one-sided tests) and posterior odds are depicted in Table 2. Note that *M* reports a point estimate based on the resulting posterior distribution and can therefore differ from the empirical means. For all interaction coefficients, $B{F}_{10}$ was smaller than 0.3 indicating that the data was more likely under the ${H}_{0}$, that is, that the regression coefficient is not different from zero. For the main effects, all $B{F}_{-0}$ but one were larger than 10 indicating that the data was more likely under ${H}_{1}$, that is, that the regression coefficient is smaller than zero. For the regression coefficient for loudness in the violin condition, $B{F}_{-0}$ was smaller than 3 and therefore indicated that data was 2.47 times more likely under the${H}_{1}$. Additional robustness checks further showed that the Bayes Factors remained comparable for a range of different prior widths (see “Appendix” for the full robustness check).

**Table 2:** Results from the Bayesian one-sample *t*-tests for the overall condition as well as separated for each timbre

Condition	Predictor	*M*	95% HDI	*BF*_10_/*BF*_−0_	Prior odds_corr_	Posterior odds_corr_
Violin	Interaction	0.01	[− 0.04, 0.05]	0.19	0.26	0.05
Overall	Interaction	− 0.01	[− 0.06, 0.04]	0.19	0.26	0.05
Organ	Interaction	− 0.01	[− 0.04, 0.02]	0.28	0.26	0.07
Violin	Loudness	− 0.05	[− 0.10, 0.00]	2.47	0.1	0.25
Organ	Loudness	− 0.07	[− 0.11, − 0.02]	16.6	0.1	1.67
Overall	Loudness	− 0.11	[− 0.19, − 0.04]	24.2	0.1	2.43
Organ	Frequency	− 0.12	[− 0.19, − 0.05]	45.2	0.1	4.55
Overall	Frequency	− 0.23	[− 0.36, − 0.11]	138	0.1	13.88
Violin	Frequency	− 0.13	[− 0.20, − 0.06]	146	0.1	14.70

HDI = Highest Density Interval. Estimation errors of the Bayes factors were all smaller than 5% and are therefore not reported. Prior odds and posterior odds are based on the corrected prior probability for *H*_0*i*_

To investigate whether musical training or perceptual abilities had an influence on the SPARC effect or the SLARC effect, we calculated Pearson’s correlation coefficients between the mean score for each scale and the overall regression coefficients. Mean scores were ${M}_{perception}=5.11$ ($SD=0.81$) and ${M}_{training}=3.20$ ($SD=1.58$). There was a significant correlation between mean scores for both scales, $r=0.70$, 95% CI $\left[0.49, 0.84\right]$, $t\left(34\right)=5.76$, $p<0.001$. Neither the mean score of perception nor the mean score of musical training did significantly correlate with any of the regression coefficients (ps > 0.05). Therefore, we resigned from further analysis.

## Discussion

The first aim of this study was to investigate whether the SPARC effect and the SLARC effect occur simultaneously in a timbre discrimination task, that is, when loudness and pitch are irrelevant for the task. Indeed, loudness as well as pitch interacted with response side: Participants responded faster to high and loud tones when responding with the top-sided response key compared to the bottom-sided response key and vice versa for soft and low tones. The dRT analyses further revealed, that mean dRT linearly decreased with increasing loudness as well as with increasing pitch. These results show that both the SPARC effect and the SLARC effect occurred, supporting our first hypothesis regarding the simultaneous occurrence of both effects. A second aim of this study was the investigation of a potential interrelation between the SPARC effect and the SLARC effect indicated by an interaction between both effects. Contrary to our second hypothesis, the predictors loudness and pitch did not interact in the dRT analyses and the effects were purely additive.

Previous studies investigated either the SLARC effect or the SPARC effect in a timbre discrimination task (Koch et al., 2023; Lega et al., 2020; Lidji et al., 2007; Rusconi et al., 2006). The results from our study did not only replicate these effects, but also showed that both effects can occur simultaneously. Our SPARC effect was numerically smaller compared to results from other studies. This can easily be explained by the use of a limited pitch range in our experiment compared to the pitch ranges used in previous studies (e.g. Lidji et al., 2007; Rusconi et al., 2006). As loudness and pitch were both task-irrelevant, the results indicate a simultaneous and automatic activation of the spatial information in both dimensions. Additionally, the continuous linear decrease of dRT with increasing loudness level indicates a continuous spatial representation rather than a categorization as it would be predicted by, for example, the polarity correspondence principle (Proctor & Cho, 2006).

The occurrence of SARC effects even when the corresponding dimension is not relevant for the task, is generally considered an indication of automatic activation of the implicit spatial information for the corresponding dimension (Dehaene et al., 1993; Weis et al., 2015). However, the use of bimanual responses may induce a spatial bias, and the processing of the spatial information would no longer be considered implicit (Shaki & Fischer, 2018; Sixtus et al., 2019). These studies used non-lateralized responses, meaning participants responded with a single response key in a go/no-go task, and therefore processed the magnitude information and spatial information implicitly. In these paradigms, participants did not respond faster when the number magnitude and horizontal spatial information matched. Therefore, it was interpreted that the horizontal SNARC effect may not reflect a spatial representation but rather a spatial processing bias. In contrast to the horizontal spatial information, a reaction time benefit was observed when number magnitude and vertical spatial information matched. This suggests that the vertical association may be inherently linked to the concept of magnitude (Shaki & Fischer, 2018; Sixtus et al., 2019). Regarding pitch and loudness, a next step may be to investigate, whether the vertical associations of these auditory dimensions still occur, when the spatial information is processed implicitly in a setting with non-lateralized responses.

The results show that the SLARC effect can indeed occur simultaneously with another SARC effect, in this case the SPARC effect. The question remains why this was not the case when investigating the SLARC effect and the SNARC effect (Hartmann & Mast, 2017). One possible explanation might be an influence of the task. Fischer et al. (2013) argued, that in the case of two competing dimensions, a potential SARC effect might only arise for the dimension which is relevant for the task. Although loudness and number magnitude are both irrelevant in a parity judgment task as used by Hartmann and Mast (2017), parity is stronger related to the numerical value of a number than to loudness. However, in the present experiment, the task-relevant dimension timbre might be equally strong related to pitch and loudness and therefore a comparable strong automatic activation of the spatial information in both dimensions might have been possible. On the other hand, Weis and colleagues (2015, 2016) also used a parity judgment task and did find simultaneous SARC effects.

For our study, separate frequentist and Bayesian analyses showed that timbre had only a negligible influence on the SPARC and SLARC effect: Both effects occurred in most of the timbre conditions. Nevertheless, the interaction patterns involving timbre partially differed for pitch and loudness. There was a significant interaction between timbre and pitch with shorter reaction times for high violin and low organ tones compared to low violin and high organ tones. This interaction pattern is comparable to the timbre-pitch interaction found by Melara and Marks (1990). Loudness and timbre did not interact in our study, indicating a slightly different influence of timbre on the processing of pitch and loudness. However, even though timbre might not be completely equally related to pitch and loudness in our study, it did not influence the SPARC and SLARC effect.

Another explanation for discrepancies with regard to the simultaneous occurrences of SARC effect could be that SARC effects for prothetic dimensions do not occur simultaneously in general. Previous studies suggest that this is at least the case for the SNARC effect and the SARC effect for physical size (Vellan & Leth-Steensen, 2022; Weis et al., 2018). However, as these studies used either a number or a size discrimination task, the non-simultaneous occurrence might be due to the different relevance of the dimensions for the task (Fischer et al., 2013). Therefore, and because empirical evidence of concurrent SARC effects is rare, this explanation should be taken with caution. Further research is needed on the simultaneous occurrence of different SARC effects and how this relates to metathetic and prothetic dimensions.

The interaction between the SPARC effect and SNARC effect found by prior studies (Fischer et al., 2013; Weis et al., 2015, 2016) did not generalize to the SLARC effect in our study. This indicates that the interaction between the SPARC effect and the SNARC effect was not due to a shared representation in the sense of ATOM as some authors suggested (Weis et al., 2016). If this would have been the case, the interaction should have generalized to the SLARC effect, as loudness is suggested to be represented as a magnitude in the sense of ATOM (Bruzzi et al., 2017; Hartmann & Mast, 2017). Instead, other mechanisms might have been responsible for the interdependence between the SPARC effect and SNARC effect, for example, sharing a common central processes as already mentioned by Weis et al. (2016).

The lack of an interaction between the SPARC and the SLARC effect supports the assumption that loudness and pitch are represented separately. In addition, the continuous linear decrease of dRT with increasing pitch and loudness indicates that both distinct representations may be continuous. According to Lidji et al. (2007), the SPARC effect may rely on a spatial representation as proposed in former representational models of musical pitch (Shepard, 1982; Ueda & Ohgushi, 1987); while Bruzzi et al. (2017) suggest that the SLARC effect is due to a generalized magnitude representation of loudness according to ATOM (Walsh, 2003).

Models aiming to describe the mental representation of pitch assume that pitch is represented spatially on a helix structure (Shepard, 1982; Ueda & Ohgushi, 1987). This assumption takes into account that an increase in frequency does not only lead to an increase in perceived pitch height but also to a change of the perceived pitch chroma. Two pitches with the same pitch chroma but from different octaves, for example C4 (261 Hz) and C5 (523 Hz), are considered subjectively more similar than two pitches with different chromas but closer frequencies, such as C4 (261 Hz) and F4 (349 Hz). Nevertheless, while pitch chroma is assumed to be represented circular, the helix structure comprises a constant vertical increase in pitch height. Therefore, even musical tones with the same pitch chroma differ in their pitch height. Thus, a spatial helix representation would still predict a continuous decrease of dRT with increasing frequency, similar to the result pattern in the current study.

A continuous decrease of dRT would be also in line with the assumption of a one-dimensional, linear spatial representation of pitch, comparable to the representation of loudness. However, this representation would not be considered a magnitude representation according to ATOM, because pitch is a metathetic dimension (Stevens, 1957; Stevens & Galanter, 1957), and therefore not part of the generalized magnitude representation system according to ATOM (Walsh, 2003, 2015). Nevertheless, the question whether the SPARC effect relies on a helix structure, or another one-dimensional spatial representation remains, and results from the current study do not allow to distinguish these spatial representation structures. Future studies should address the question whether reaction times indicating a SPARC effect also indicate a spatially organized helix structure of the underlying representation, for example by taking into account the influence of pitch similarity on reaction times in same-different judgments (Cohen Kadosh et al., 2008).

In contrast to pitch, loudness may be represented as a magnitude in the sense of ATOM (Bueti & Walsh, 2009; Walsh, 2003). In this case, the SLARC effect would be an instance of the more general SQUARC effect. This assumption is supported by the prothetic character of loudness (Stevens, 1957; Stevens & Galanter, 1957) and by interactions between loudness and other ATOM-related magnitudes (Alards-Tomalin et al., 2015; Hartmann & Mast, 2017; Heinemann et al., 2013; Takeshima & Gyoba, 2013). The question remains whether a vertical SLARC effect is in line with this interpretation. The direction of the spatial association in the context of ATOM is not narrowed to the horizontal dimension. Furthermore, it is assumed that numbers—one of ATOM’s most prominent quantity dimension—are also spatially represented in the vertical dimension (Aleotti et al., 2023; Ito & Hatta, 2004; see Winter et al., 2015 for a review). This vertical spatial association might be present in other magnitudes as well.

In conclusion, our study has shown that the SPARC effect and the SLARC effect occur simultaneously, but appear to be independent of each other. This supports the interpretation that both effects are due to separate spatial representations. Furthermore, our study extended the findings on simultaneous SARC effects and showed that the implicit spatial information of two dimensions can be automatically activated simultaneously. Whether and how this is influenced by task characteristics or by specific characteristics of the dimensions (e. g. the distinction between prothetic and metathetic dimensions) needs to be investigated in further research. In addition, future research could help to understand the complex patterns of interaction between different SARC effects and what leads to interdependencies between spatial associations in different dimensions.

## Figures and Tables

**Fig. 5:**
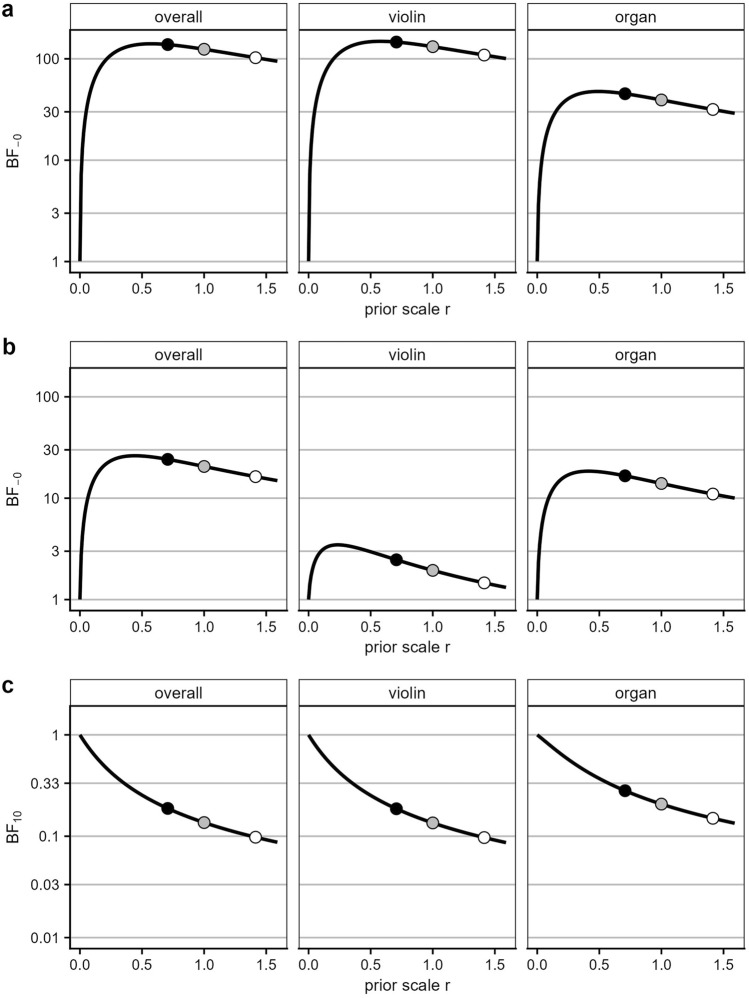
Bayes Factors as a function of prior scale of the Cauchy distribution for the regression coefficients of pitch (**a**), loudness (**b**), and interaction (**c**). *Note*: Points indicate Bayes Factors at prior scale ${\text{r}} = \sqrt {1/2}$ (black), r = 1 (grey), and ${\text{r}} = \sqrt {2}$ (white)

## Data Availability

The data generated and/or analysed during the current study are available from the corresponding author on reasonable request.

## Appendix

See Fig. 5.

## Footnotes

1 Please note, that the term SLARC effect is also used in the context of spatial associations for linguistic material (Abbondanza et al., 2021).

2 Please note, that the approach for one-sided leads to two rival hypotheses (van Doorn et al., 2020).
